# Evaluation and Design of Colored Silicon Nanoparticle Systems Using a Bidirectional Deep Neural Network

**DOI:** 10.3390/nano12152715

**Published:** 2022-08-07

**Authors:** Yan Zhou, Lechuan Hu, Chengchao Wang, Lanxin Ma

**Affiliations:** 1 School of Energy and Power Engineering, Shandong University, Jinan 250061, China; 2 Optics & Thermal Radiation Research Center, Institute of Frontier and Interdisciplinary Science, Shandong University, Qingdao 266237, China

**Keywords:** silicon nanoparticles, structural color, Lorentz–Mie theory, deep neural networks, Monte Carlo simulations

## Abstract

Silicon nanoparticles (SiNPs) with lowest-order Mie resonance produce non-iridescent and non-fading vivid structural colors in the visible range. However, the strong wavelength dependence of the radiation pattern and dielectric function makes it very difficult to design nanoparticle systems with the desired colors. Most existing studies focus on monodisperse nanoparticle systems, which are unsuitable for practical applications. This study combined the Lorentz–Mie theory, Monte Carlo, and deep neural networks to evaluate and design colored SiNP systems. The effects of the host medium and particle size distribution on the optical and color properties of the SiNP systems were investigated. A bidirectional deep neural network achieved accurate prediction and inverse design of structural colors. The results demonstrated that the particle size distribution flattened the Mie resonance peak and influenced the reflectance and brightness of the SiNP system. The SiNPs generated vivid colors in all three of the host media. Meanwhile, our proposed neural network model achieved a near-perfect prediction of colors with high accuracy of the designed geometric parameters. This work accurately and efficiently evaluates and designs the optical and color properties of SiNP systems, thus accelerating the design process and contributing to the practical production design of color inks, decoration, and printing.

## 1. Introduction

Structural color generation from high-refractive-index dielectric nanostructures connects color organisms in nature with the rapid emergence of nanophotonic coloring technology. The structural color produced by the light–matter interaction in the nanostructure is better than traditional color in many aspects. It does not fade as long as the structure remains unchanged. Therefore, it is widely used in color printing, decoration, and anti-counterfeiting [1,2,3,4,5]. To avoid iridescence caused by Bragg diffraction, nanostructures of high-refractive-index dielectrics in the visible range have been widely studied in recent years [6,7,8]. High-refractive-index dielectric nanoparticles based on a relatively sharp electric dipole (ED) and magnetic dipole (MD) Mie resonance, which can generate non-fading and non-iridescent high-resolution structural colors [9,10,11,12,13], have emerged as an alternative to plasmonic nanostructures. Moreover, compared to the plasmonic nanostructures based on the localized surface plasmon resonance, the high-refractive-index dielectric nanostructure is less costly. Its resonance wavelength depends strongly on the size of the nanostructure [14,15].

High-refractive-index dielectric materials, such as silicon (Si), titanium dioxide (TiO_2_), and germanium (Ge), have attracted widespread attention owing to their excellent optical properties. Among these, silicon nanoparticles (SiNPs) with a particle size range of 100–200 nm have been studied most intensively [16,17,18]. Sugimoto et al. [6] employed silicon monoxide (SiO) powder as a raw material to prepare high-sphericity silicon nanospheres-structured color ink for vivid color. The color inks of silicon nanospheres combined with a polymer binder can color flexible substrates via a painting process. Flauraud et al. [11] optimized a silicon nanodisc array to fabricate high-resolution color features and millimetric painting replicas. They discussed high-throughput electron beam lithography and hybrid color elements. This work paved the way for the broader exploitation of nanoscale color printing. Okazaki et al. [16] presented a process to control the hue, saturation, and brightness of SiNPs inks and demonstrated that the reflection color is determined by the balance of Kerker-type backward scattering and multiple scattering. In addition, they developed a SiNP ink mixed with carbon black to realize vivid reflection colors under room light. Dong et al. [19] designed a novel nanostructure consisting of silicon and silicon nitride. It can expand the color gamut while maintaining the print resolution. This nanostructure design achieves a color gamut superior to that of sRGB and is compatible with CMOS processes.

Based on the above research on SiNPs, the structural color of the nanoparticle system depends on the material type, particle-size distribution, radius, volume fraction, and background medium. Therefore, by designing these parameters, the relevant structural colors can be adjusted. At present, the conventional design task mainly depends on finite difference time domain (FDTD) [20,21], particle swarm optimization [22,23], genetic algorithm, and the trial-and-error method [24,25,26]. They involve the convergence of the initial random design to the desired design through continuous optimization. In order to ensure the needs of practical applications, multiple structural parameters should usually be adjusted to generate thousands of colors. However, for structures with mixed shape, size, and other design parameters, the convergence speed of the above method decreases significantly with the increase of the complexity of structures, so these methods may be unsuitable for the structural color design of complex micro/nanostructures. Fortunately, the unprecedented development of machine learning has made it a powerful tool for solving complex computing and inverse design problems. Several studies have reported machine learning methods to facilitate the design of structural colors and inverse design of nanostructures and materials to achieve the desired optical response [27,28,29,30,31,32,33]. So et al. [28] achieved the simultaneous inverse design of materials and structural parameters of core–shell nanoparticles by using neural networks. In addition, they developed a neural network to discover spectrally isolated pure magnetic dipole resonances and spectrally overlapping electric and magnetic dipoles. Dai et al. [30] first reported a bidirectional artificial neural network to inversely design the Fabry–Perot cavity structure parameters for deep learning. Its range of color space coverage was 215% wider than sRGB. The bidirectional artificial neural network was first proposed by Liu et al. [34]. It solves the problem caused by non-uniqueness in all inverse scattering problems and paves the way for deep neural networks to design complex nanophotonic structures.

The studies mentioned above have made significant progress in the structural color design of micro/nanostructured materials, especially for SiNP systems. However, the efficient evaluation and design of nanoparticle systems remains challenging when numerous factors, such as the particle size distribution and host medium, are considered. In this study, the color and radiative properties of polydisperse SiNPs embedded in three different host media were systematically investigated. A bidirectional deep neural network was established to accurately predict the color properties of SiNP systems and inversely design the geometric parameters for the desired colors. This study provides a simple and convenient method to design the structural colors of SiNP systems accurately and efficiently, as is beneficial for practical applications of color printing, decoration, and inks.

## 2. Model and Methods

**Figure 1:**
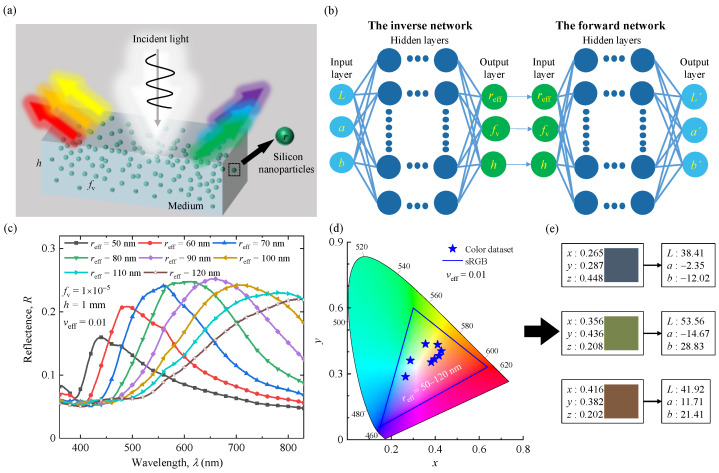
Overall process of evaluating and designing the parameters of SiNP systems. (**a**) Schematic diagram of the radiative transfer process of the SiNP system. (**b**) The architecture of a bidirectional neural network for predicting colors and design geometry parameters of SiNPs system. (**c**) The reflectance spectra of SiNP systems for different geometric parameters with *h* = 1 mm and *f*_v_ = 1 × 10^−5^. (**d**,**e**) The color generated by dispersing SiNP system in water in CIE-1931-XYZ and CIE-1976-Lab chromaticity diagram.

Our theoretical evaluation and design process for the structural colors of silicon nanoparticle systems are represented schematically in Figure 1. The simulation domain consists of a thin medium layer containing SiNPs (Figure 1a). It can be viewed as a typical model of colloidal suspensions and nanocomposite coatings. In this work, three different background media (water, polydimethylsiloxane (PDMS), and polymethyl methacrylate (PMMA)), four geometric parameters (particle effective radius, *r*_eff_; particle volume fraction, *f*_v_; effective variance, *v*_eff_; and layer thickness, *h*), and color properties (*L*, *a*, and *b*) in the CIE-1976 color space are significant features for evaluating and designing colored SiNP systems. The optical and color properties of SiNP systems were obtained from extensive simulation processes, including Mie scattering calculations, Monte Carlo simulations, and spectrum-to-color conversion, as shown in Figure 1c–e. We then created a bidirectional deep neural network to predict the generated colors of SiNP systems and inverse design the geometric parameters for the desired colors, as depicted in Figure 1b. In general, SiNPs have good potential application prospects in regard to structural color.

### 2.1. Optical Properties of a Single Nanoparticle

The optical properties of single nanoparticles should be determined before solving the radiative transfer problem of the nanoparticle systems. An isolated spherical particle with radius, *r*, and complex refractive index is solved by using the Lorentz–Mie theory [35,36]. Therefore, the scattering and extinction cross-sections can be obtained by using the following equations [35,36]:

(1) $$C_{\mathrm{sca}}=\frac{2\pi }{k_{1}{}^{2}}\overset{\infty }{\underset{n=1}{\sum }}\left(2n+1\right)\left({\left|a_{n}\right|}^{2}+{\left|b_{n}\right|}^{2}\right)$$

(2) Cext=2πk12∑n=1∞2n+1Rean+bn

where *k*_1_ = 2π*n*_m_/*λ* is the wave number in the host medium, *n*_m_ is real part of refractive index of the host medium, and *a_n_* and *b_n_* are the Mie coefficients.

The scattering phase function shows the spatial distribution of scattering energy, which is calculated as follows [35,36]:

(3) $$\Phi_{p}\left(\theta \right)=\frac{2\pi }{C_{\mathrm{sca}}}\left[{\left|S_{11}\left(\theta \right)\right|}^{2}+{\left|S_{22}\left(\theta \right)\right|}^{2}\right]$$

where *S*_11_ and *S*_22_ are the amplitude scattering matrix elements.

For the polydisperse SiNP system, the nanoparticle size distributions obey some statistical laws. Here, the conventional gamma distribution was applied to represent the particle size distribution of the SiNPs. The gamma distribution function, *n*(*r*), is as described by Hansen and Travis [37]:

(4) $$n\left(r\right)=\mathrm{constant}\times r^{\frac{\left(1-3b\right)}{b}}\times exp\left(\frac{-r}{ab}\right),b\in \left(0,0.5\right)$$

(5) $$r_{\mathrm{eff}}=\frac{1}{{\left\langle G\right\rangle }_{r}}\int_{r_{\min}}^{r_{\max}}\pi r^{3}n\left(r\right)dr$$

(6) $$\nu_{\mathrm{eff}}=\frac{1}{{\left\langle G\right\rangle }_{r}r_{\mathrm{eff}}^{2}}\int_{r_{\min}}^{r_{\max}}{\left(r-r_{\mathrm{eff}}\right)}^{2}\pi r^{2}n\left(r\right)dr$$

where *a* and *b* correspond to effective radius, *r*_eff_, and effective variance, *v*_eff_, when *r*_min_ = 0 and *r*_max_ = ∞. The size distribution with *v*_eff_ = 0 corresponds to monodisperse situations, and ${\left\langle G\right\rangle }_{r}$ represents the average area of the geometric projection of each particle. The ensemble-averaged extinction and scattering coefficient factor per particle can be calculated as follows [36]:

(7) $${\langle C}_{\mathrm{sca}\rangle }{}_{r}=\int_{r_{\min}}^{r_{\max}}C_{\mathrm{sca}}\left(r\right)n\left(r\right)dr\approx \overset{N_{r}}{\underset{i=1}{\sum }}u_{i}C_{\mathrm{sca}}\left(r_{i}\right)n\left(r_{i}\right)$$

(8) $${\langle C}_{\mathrm{ext}\rangle }{}_{r}=\int_{r_{\min}}^{r_{\max}}C_{\mathrm{ext}}\left(r\right)n\left(r\right)dr\approx \overset{N_{r}}{\underset{i=1}{\sum }}u_{i}C_{\mathrm{ext}}\left(r_{i}\right)n\left(r_{i}\right)$$

where *r_i_* and *u_i_* are the division points and weights of the quadrature formula, respectively, in the interval [*r*_min_*, r*_max_], and *N_r_* is the number of quadrature division points.

### 2.2. Optical Properties of Nanoparticle Systems

For dilute SiNP systems, the total radiative properties of the SiNP system can be expressed as follows [36]:

(9) $$\mu_{\mathrm{sca}}=\mu_{\mathrm{sca},p}=n_{0}\int_{r_{\min}}^{r_{\max}}n\left(r\right)C_{\mathrm{sca}}\left(r\right)dr=\frac{f_{v}}{{\left\langle V\right\rangle }_{r}}{\langle C}_{\mathrm{sca}\rangle }{}_{r}$$

(10) $$\mu_{\mathrm{ext}}=\mu_{\mathrm{ext},p}+\mu_{\mathrm{ext},m}=\frac{f_{v}}{{\left\langle V\right\rangle }_{r}}{\langle C}_{\mathrm{ext}\rangle }{}_{r}+\mu_{\mathrm{ext},m}$$

(11) $$\Phi \left(\theta \right)=\frac{n_{0}}{\mu_{\mathrm{sca}}}\int_{r_{\min}}^{r_{\max}}\Phi_{p}\left(r,\theta \right)n\left(r\right)C_{\mathrm{sca}}\left(r\right)dr$$

where *μ*_sca_, *μ*_ext_, and Φ(*θ*) are the scattering coefficient, extinction coefficient, and scattering phase function of the SiNP system, respectively; *μ*_sca,p_ and *μ*_ext,p_ are the scattering coefficient and extinction coefficient of the particles; *μ*_ext,m_ *=* 4π*κ*_m_/*λ* is the extinction coefficient of host medium; *κ*_m_ is the imaginary part of refractive index of the host medium; 〈*V*〉*_r_* is the average volume per particle; and *f*_v_ is the volume fraction of silicon nanoparticles. In addition, the dielectric function of SiNPs was taken from Aspnes and Studna’s dataset in 1983 [38]. The complex refractive index of a pure medium (water [39]; PMMA and PDMS [40,41]) was used.

### 2.3. Monte Carlo Simulation of Radiative Transfer Process

The Monte Carlo calculation model is illustrated in Figure 1a. In the model, the range of *r*_eff_ was set to be 50 to 120 nm with 2 nm intervals. The range of *f*_v_ was set to be 5.0 × 10^−6^ to 1.0 × 10^−4^ with 5.0 × 10^−6^ intervals. The range of *h* was set to 0.5ߝ10 mm in 0.5 mm intervals. The effective variance, *v*_eff_, was set to 0, 0.01, and 0.05, respectively. The external medium of the system was air.

To understand the multiple scattering behavior of monodisperse and polydisperse SiNP systems and predict their reflection colors, we designed a Monte Carlo–based computational package simulation [42,43]. For known radiative properties of the sparsely dispersed medium, the radiative energy transfer can be computed by solving the radiative transfer equation (RTE). It is written as follows [44]:

(12) $$s\cdot \nabla I=-\mu_{\mathrm{ext}}I+\frac{\mu_{\mathrm{sca}}}{4\pi }\int_{4\pi }I\left(\Omega^{\prime }\right)\Phi \left(\Omega^{\prime },\Omega \right)d\;\Omega^{\prime }$$

where *I* is the radiation intensity along the propagation direction, **s**. An infinitely thin light beam is perpendicularly incident on the upper boundary of the layer by default. After interaction with the layer, the reflected photons are collected. The directional-hemispherical reflectance, *R*, of the layer is determined from the following [44]:

(13) $$R=\frac{\sum_{2\pi }N_{\mathrm{ref}}}{N_{0}}$$

where *N*_0_ is the total number of photons that are incident on the layer. *N*_ref_ is the number of photons that are collected using detectors positioned in the hemispherical space outside the upper surface.

### 2.4. Spectrum to Color Conversion

Color is a subjective perception of the observer rather than a property of electromagnetic radiation. Therefore, the obtained reflectance spectra must be transformed into the corresponding color coordinates in the color space. The CIE-1976-Lab and CIE-1931-XYZ color spaces are typically utilized to evaluate the colors generated by nanoparticle systems. The color coordinates of the CIE-1931-XYZ color space can be calculated as follows [45,46]:

(14) $$X=100\frac{\int I_{D65}\left(\lambda \right)R\left(\lambda \right)\overset{¯}{x}\left(\lambda \right)d\lambda }{\int I_{D65}\left(\lambda \right)\overset{¯}{y}\left(\lambda \right)d\lambda }$$

(15) $$Y=100\frac{\int I_{D65}\left(\lambda \right)R\left(\lambda \right)\overset{¯}{y}\left(\lambda \right)d\lambda }{\int I_{D65}\left(\lambda \right)\overset{¯}{y}\left(\lambda \right)d\lambda }$$

(16) $$Z=100\frac{\int I_{D65}\left(\lambda \right)R\left(\lambda \right)\overset{¯}{z}\left(\lambda \right)d\lambda }{\int I_{D65}\left(\lambda \right)\overset{¯}{y}\left(\lambda \right)d\lambda }$$

where *I*_D65_(*λ*) is the spectral power distribution of the standard D_65_ illuminance; $\overset{¯}{x}\left(\lambda \right)$, $\overset{¯}{y}\left(\lambda \right)$, and $\overset{¯}{z}\left(\lambda \right)$ are the spectral tristimulus values that contain information about the light source used. The chromaticity coordinates *x* and *y* were determined by using the following normalized parameters [45]:

(17) $$x=\frac{X}{X+Y+Z}$$

(18) $$y=\frac{Y}{X+Y+Z}$$

(19) $$z=\frac{Z}{X+Y+Z}$$

Normalization parameter matched the corresponding chromaticity coordinates in the color space. The CIE-1976-Lab color space is more homogeneous and closely corresponds to CIE-1931-XYZ. Therefore, it is more suitable as a color space for identifying color differences. The CIE-1976-Lab color space is defined by three tristimulus values *L*, *a*, and *b*. *L* represents color brightness, *a* stands for redness (+) and greenness (−), and *b* represents yellowness (+) and blueness (−). The conversion functions between (*X*, *Y*, and *Z*) and (*L*, *a*, and *b*) are as follows [47]:

(20) $$L=116f\left(\frac{Y}{Y_{n}}\right)-16$$

(21) $$a=500\left[f\left(\frac{X}{X_{n}}\right)-f\left(\frac{Y}{Y_{n}}\right)\right]$$

(22) $$b=200\left[f\left(\frac{Y}{Y_{n}}\right)-f\left(\frac{Z}{Z_{n}}\right)\right]$$

with *X_n_*, *Y_n_*, and *Z_n_* being the tristimulus values of a reference white object:

(23) $$f\left(s\right)=s^{\frac{1}{3}}\;\mathrm{if}\;s>{\left(\frac{24}{116}\right)}^{3}$$

(24) $$f\left(s\right)=\left(\frac{841}{108}\right)s+\frac{16}{116}\;\mathrm{if}\;s\leq {\left(\frac{24}{116}\right)}^{3}$$

Δ*E*_1976_ fits well with the way human observers perceive small color differences. Hence, the three tristimulus values *L*, *a* and *b* are more suitable for the quantitative comparison of color than *X*, *Y*, and *Z*. The CIE color-difference function, Δ*E*, can be defined as the Euclidean distance between two color vectors (*L*, *a*, and *b*) and (*L*′, *a*′, and *b*′) [47]:

(25) $$\Delta E=\sqrt{{\left(L^{\prime }-L\right)}^{2}+{\left(a^{\prime }-a\right)}^{2}+{\left(b^{\prime }-b\right)}^{2}}$$

### 2.5. Deep Neural Network Framework

A schematic diagram of this bidirectional neural network model is shown in Figure 1b. It consists of forward and inverse networks. The backward network is connected in series with the trained forward network [34]. Subsequently, a large amount of normalized training set data was input into the forward neural network for training. In the training process, considering the geometric parameters, the forward neural network can accurately predict the structural color.

In the inverse design, we input the desired color parameters into the inverse neural network for training and predicting the corresponding geometric parameters. Owing to the non-uniqueness of the predicted results, we input the predicted geometric parameters into the previously trained forward neural network to obtain the corresponding color parameters. We further evaluate the performance of the inverse neural network, using the mean squared difference between the two calculated types of color parameters. Further-more, the numbers of layers and neurons in the hidden layer of neural network are determined by continuously minimizing the loss function, which is defined as the mean squared error between the predicted and true *Lab* values:

(26) $${\mathrm{MSE}}_{Lab}=\frac{1}{N}\overset{N}{\underset{i=1}{\sum }}{\left(L_{\mathrm{predicted}}^{\prime }-L_{\mathrm{true}}\right)}^{2}+{\left(a_{\mathrm{predicted}}^{\prime }-a_{\mathrm{true}}\right)}^{2}+{\left(b_{\mathrm{predicted}}^{\prime }-b_{\mathrm{true}}\right)}^{2}$$

where *N* is the total number of datasets. The loss function notably compares the parameters of the CIE-1976 color space (*Lab*) rather than the geometric parameters. The loss function determines the accuracy of the prediction. Therefore, in an inhomogeneous CIE-1931-XYZ space, identical Euclidean distances between XYZ vectors may mean different color differences, leading to bias optimization for some colors [48]. In the CIE 1976-Lab space, the same Euclidean distance represents the same chromatic aberration with a higher design accuracy.

## 3. Results and Discussion

### 3.1. Effects of Particle Size and Distribution on SiNP Systems

**Figure 2:**
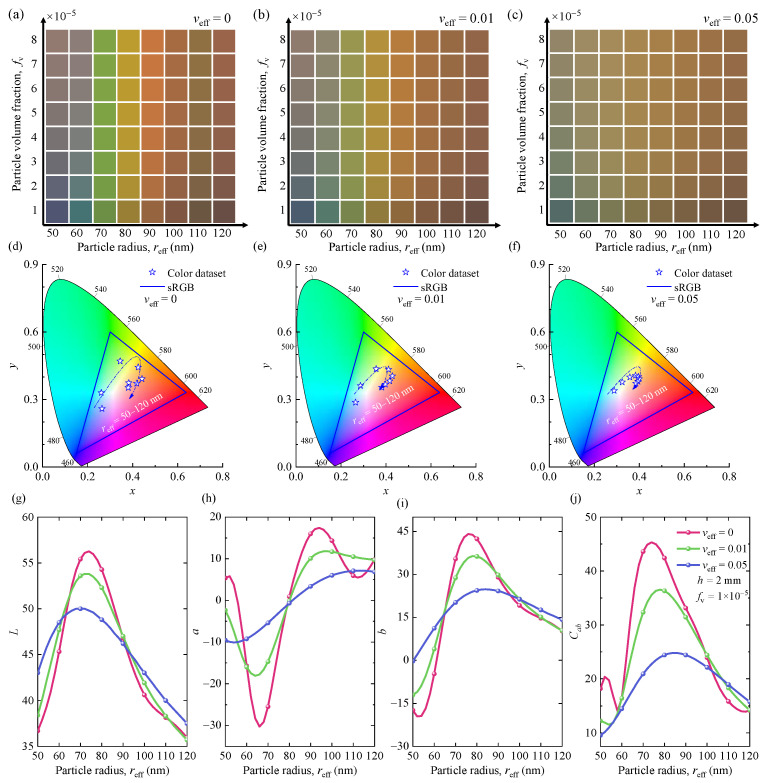
(**a**–**c**) Structural colors of SiNP systems (medium is water) for different geometric parameters with *h* = 2.0 mm. (**d**–**f**) The corresponding chromaticity diagram (CIE-1931) of the SiNPs for different particle sizes and distributions with *h* = 2.0 mm and *f*_v_ = 1.0 × 10^−5^. (**g**–**j**) The color properties (*L*, *a*, *b*, and *C*_ab_) correspond to different particle radii and effective variances.

**Figure 3:**
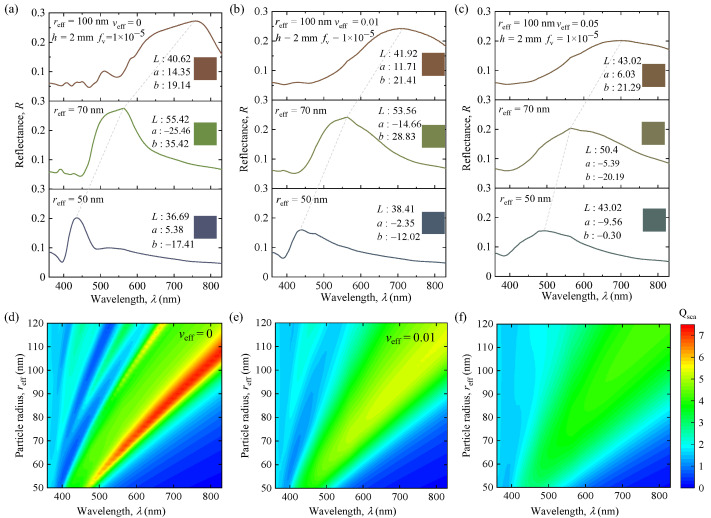
(**a**−**c**) The reflectance spectra of SiNP systems (medium is water) with different particle sizes and distributions. (**d**−**f**) Represent the scattering efficiency factors of polydisperse SiNPs with *v*_eff_ = 0, 0.01, and 0.05, respectively.

Geometric and structural parameters, such as the particle radius, volume fraction, thickness, surface condition of the layer, and dispersion of the particle system, are significant for the optical properties of nanoparticle systems. To clarify the relationship among different influencing parameters, especially particle size and size distribution, the optical and color properties of monodisperse and polydisperse SiNPs embedded in water are discussed in Figure 2 and Figure 3.

Figure 2a–c presents the vivid generated colors for different particle effective radii, volume fractions, and effective variances of the SiNP system. Figure 2d–f shows the corresponding chromaticity diagrams (CIE-1931) of SiNP systems with different effective radii and effective variances. The specific color coordinates corresponding to each color are shown in Supplementary Figure S1. To facilitate the analysis, the color properties *L*, *a*, *b*, and *C*_ab_ of SiNP systems are also illustrated in Figure 2g–j. The combination of *a* and *b* determines its chromaticity, which includes the saturation $C_{\mathrm{ab}}=\sqrt{a^{2}+b^{2}}$ of the color. As shown, the generated colors of monodisperse SiNPs embedded in water are affected by the particle effective radii and volume fractions. As the particle effective radius increases, the colors generated by the SiNP system change from blue to green, and then from orange to red. Meanwhile, the particle size has a greater influence on chromaticity. The volume fraction only changes the lightness of the color. In the polydisperse SiNP system, increasing the effective variance fades the corresponding color. The color gamut in the CIE-1931 color space tends to be white. These phenomena can be explained by the variations of color properties *L*, *a*, *b,* and *C*_ab_, as shown in Figure 2g–j. For example, under three different *v*_eff_, the lightness, *L*, increases at first with the increasing *r* and then decreases after reaching the maximum value. As the *v*_eff_ increases, the range of variation of redness and greenness, *a*, and blueness and yellowness, *b*, gradually decreases. It proves that the color gamut in the CIE-1931 color space of SiNP systems gradually decreases, as shown in Figure 2h,i. In addition, *v*_eff_ also affects the saturation, *C*_ab_, of the generated color of the SiNP system. For example, in SiNPs with particle radius less than 100 nm, the color saturation decreases with increasing *v*_eff_.

To facilitate understanding, the effects of particle size and distribution on the optical properties of SiNP systems are presented in Figure 3. Figure 3a–c shows the simulated reflectance spectra of the monodisperse and polydisperse SiNP systems. Figure 3d–f shows the efficiency factors of single particles for different sizes and distributions. The optical properties of the particles are closely related to the particle sizes and distributions. They are crucial for the radiative transfer of the SiNP system and influence the results of multiple scattering effects between the SiNPs. Thus, they have different degrees of influence on the optical and color properties of SiNP systems. As the particle effective radius increases, additional lowest-order scattering modes are introduced into the spectra, increasing the number of sharp scattering peaks in the visible range. It causes the resonance peaks to shift red, as shown in Figure 3a–c. This phenomenon explains the vivid color change in the monodisperse SiNP system. Meanwhile, the increase in the effective variance, *v*_eff_, smoothens the low-frequency scattering of individual particles and causes the sharp resonance peaks into smooth, broad, and few peaks, and this further leads to a significant change in the resulting colors. This is because the reflection color properties are determined by the lowest-order scattering peaks at different wavelengths. If the resonance peaks are influenced by particle sizes and distributions, the corresponding color properties will also change.

In conclusion, the particle size and distribution have a significant impact on the optical and color properties of the SiNP systems by changing the position and shape of the resonance peak. Therefore, a vivid and wide range of structural colors can be presented by controlling the geometric parameters of the SiNP system.

### 3.2. Effect of Background Media on Nanoparticle Systems

In practical applications of color inks and films containing SiNPs, different background media also affect the optical and color properties of the SiNP system. In this section, we focus on the color and optical properties of SiNP systems by using three different background mediums (i.e., water, PDMS, and PMMA).

**Figure 4:**
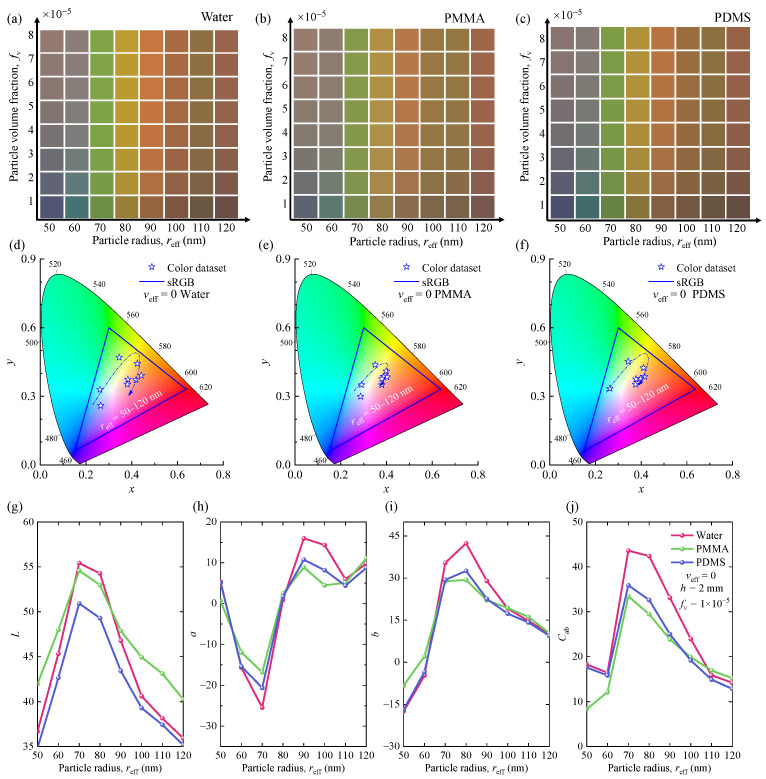
(**a**–**c**) Structural colors of monodisperse SiNP systems for different background mediums with *h* = 2.0 mm. (**d**–**f**) The corresponding chromaticity diagram (CIE-1931) of mono-disperse SiNP systems for different background mediums with *h* = 2.0 mm and *f*_v_ = 1.0 × 10^−5^. (**g**–**j**) The color properties (*L*, *a*, *b*, and *C*_ab_) correspond with different particle radii and background mediums.

Figure 4a–c illustrates the structural colors for different host media, particle radii, and particle volume fractions. Figure 4d–f shows the corresponding chromaticity diagrams (CIE-1931) of the SiNP systems with different radii and host media. The specific color coordinates corresponding to each color are shown in Supplementary Figure S2. Figure 4g–j shows the variations in the color property parameters *L*, *a*, *b*, and *C*_ab_ of the generated colors, respectively. To further investigate the effect of different host media on the generated colors and optical properties of SiNP systems, the optical constants of three different media and simulated reflection spectra of monodisperse SiNPs in different media are shown in Supplementary Figure S3. As shown, the SiNPs embedded in the three media exhibited similar vivid and colorful structural colors. This is because of the similar optical constants (*n*_m_ and *κ*_m_) of the three host media, as shown in Supplementary Figure S3b,c. For SiNPs with a radius smaller than 70 nm, corresponding color coordinates on the CIE color space shift significantly as the background medium changes. Compared to the other two media, the color gamut of the corresponding chromaticity diagram is obviously larger for the SiNPs embedded in water. Meanwhile, the values of the color hues *a* and *b* of the generated colors are significantly different for the SiNPs embedded in different media, as shown in Figure 4g–j. When the medium is water, the range of variation of redness and greenness, *a*, and blueness and yellowness, *b*, is the largest. This explains why its color gamut is the largest in the CIE color space.

Furthermore, the host medium impacts the lightness, *L*, and saturation, *C*_ab_, of the nanoparticle systems, as shown in Figure 4g–j. The lightness, *L*, of the SiNP system with three different background media increased first and decreased after the particle radius reached approximately 70 nm. The lightness increased more rapidly in water than in the other two media. As the particle size increased to approximately 70 nm, the saturation, *C*_ab_, of the SiNP system embedded in water and PDMS first decreased. It then increased rapidly to the maximum after the particle size increased to approximately 60 nm. The saturation of the SiNP system in PMMA continued to increase until the maximum. The saturation value of SiNPs embedded in water was the highest at particle radii of less than 100 nm. This is due to the multiple scattering effects of the SiNPs in different media.

As discussed above, SiNPs embedded in three different background media have similar optical properties, and different media have little effect on color properties. Therefore, they all generate vivid colors and have broad application prospects.

### 3.3. Forward Prediction and Inverse Design of Color Generation of Si Nanoparticles

A bidirectional neural network is first trained to obtain accurate color prediction based on geometric parameters. It is then used for the inverse design of the structure based on the desired color. A total of 80% of the entire dataset is used for the training set (10,944). A total of 10% is used for the validation sets (1368) and test sets (1368). To further evaluate the performance of the bidirectional neural network, we consider the example of monodisperse and polydisperse SiNPs embedded in PMMA for color prediction and structure inverse design.

**Figure 5:**
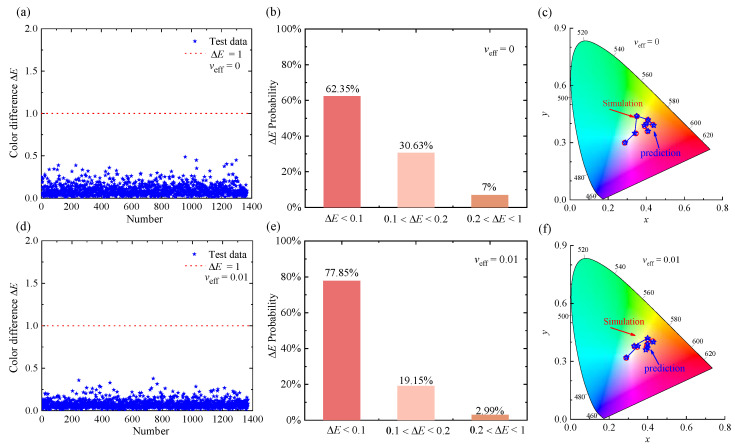
Results of the test samples in the forward network: (**a**–**c**) and (**d**–**f**) are the predicted results for monodisperse and polydisperse (*v*_eff_ = 0.01) SiNPs embedded in PMMA, respectively. (**a**,**d**) Show the color difference, Δ*E*, between the predicted and true colors. (**b**,**e**) Show the Statistical distribution of test results in different color-difference ranges. (**c**,**f**) Compare the predicted (blue pentagons) and simulated (red hollow circle) colors in the CIE−1931 color map.

The forward neural network consists of an input layer, several hidden layers, and an output layer with many neurons in each hidden layer. By minimizing the training and validation loss and continuously optimizing the structural parameters, we finally determined that the forward neural network has three hidden layers, and each hidden layer contains 200 neurons. The loss functions of the training and validation sets over the epochs are shown in Supplementary Figure S4a. It takes the parameters *r*_eff_, *f*_v_, and *h* as input and *L*, *a*, and *b* as outputs, which can be converted to other color vectors, such as sRGB, for different applications. To test the accuracy and generalization ability of the forward neural network, 1368 groups of new test data were used and analyzed. The color differences, Δ*E*, between the predicted and simulated colors and their statistical distributions are shown in Figure 5. The color-difference values, Δ*E*, are less than 1.0. It demonstrates that the forward neural network has good prediction ability. Figure 5c,f compares the color coordinates in CIE-1931 obtained by prediction and simulation. The specific color coordinates corresponding to each color are shown in Supplementary Figure S5. In this case, it is more intuitive to analyze the performance of the forward neural network. In summary, the above results indicate that a forward neural network can predict the structural color of the SiNP system with high accuracy.

**Figure 6:**
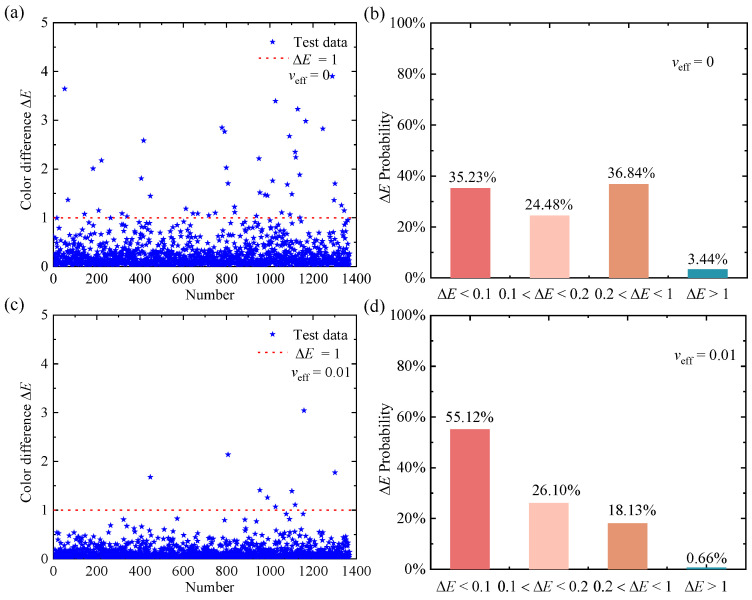
Results of the test samples in the inverse network for monodisperse and polydisperse (*v*_eff_ = 0.01) SiNP systems. (**a**,**c**) The color differences, Δ*E*, between the predicted colors and true colors and (**b**,**d**) the statistical distribution of test results in different color-difference ranges.

For the inverse design, the training of the inverse neural network is more difficult due to its non-unique nature (one color can be formed by different nanoparticle system structural parameters). This multi-solution property may pull weights to different local or global minima during the training process, making it difficult to achieve convergence during training. Therefore, the bidirectional neural network architecture with a tandem training strategy is employed to solve the multi-solution problem in this work. The output parameters (*r*_eff_, *f*_v_, and *h*) from the inverse neural network are directly input into our pretrained forward neural network to predict the color property (*L*′, *a*′, and *b*′), as shown in Figure 1b. After continuous optimization, the inverse neural network consists of four hidden layers with 100 neurons. The loss functions of training and validation sets over epoch are shown in Supplementary Figure S4b. Figure 6 shows the color-difference values, Δ*E*, of monodisperse and polydisperse (*v*_eff_ = 0.01) SiNP systems. Most of the color-difference values (monodisperse (96.56%) and polydisperse (99.34%)) are less than 1, and only a few color-difference values are larger than 1.0 for monodisperse (3.44%) and polydisperse (0.66%) SiNP systems, which also proves that our model based on the inverse neural network can accurately design the structural parameters corresponding to the target color.

**Figure 7:**
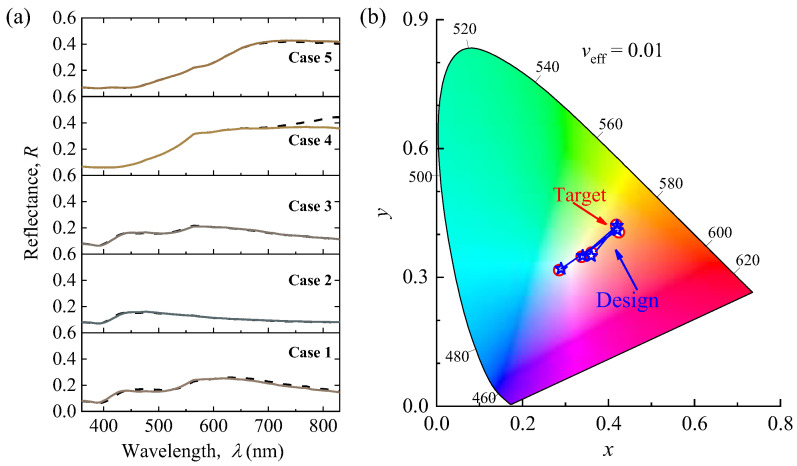
Accurate inverse designing of the geometric parameters of polydisperse (*v*_eff_ = 0.01) SiNP systems for desired colors. (**a**) Comparison of spectra between the target (color line) and design (dash line). (**b**) Comparison of design (blue pentagons) and target colors (red hollow circle) in CIE-1931 color map.

In addition, we randomly selected five groups of polydisperse (*v*_eff_ = 0.01) SiNP system test data to evaluate the performance of the inverse neural network. Figure 7a compares the target spectrum (colored line) and design spectrum (dashed line) obtained by calculation. Figure 7b compares the design color and target color coordinates in the CIE-1931. As listed in Table 1, the target values of *Lab* are then fed into the inverse neural network to obtain the design geometry parameters. After converting the design structures to design color through simulation calculation, the design results are in good agreement with the targeted results. Even if there are two cases with the color difference of Δ*E* > 1, it is still difficult for the human eyes to distinguish their color difference, and their corresponding spectra and color coordinates are very similar, thus further demonstrating the reliability and accuracy of our inverse neural network.

In summary, compared with time-consuming numerical simulation methods, our bidirectional neural network enables quick and highly accurate color prediction and structural parameter design for complex nanoparticle systems, thus greatly reducing the time and cost of color design. The detailed training process of the neural network and the process of numerical simulation are described in the Supplement Materials. This deep-learning method will be extremely beneficial for the development of nanophotonics.

## 4. Conclusions

In summary, we focused on the effects of geometrical parameters and background medium on the radiative properties and reflected color of a SiNP system through Monte Carlo and Mie scattering simulations. As the effective variance, *v*_eff_, increases, the color gamut of the SiNP systems becomes narrower, and the brightness and saturation values are also affected. When *v*_eff_ is 0.01, the effect is not significant. However, when *v*_eff_ increases to 0.05, the reflectance color and spectrum of the SiNP systems change significantly. Meanwhile, the SiNP systems embedded in water, PMMA, and PDMS all exhibit vivid colors. This indicates that SiNP systems can be widely used in the manufacturing of colored inks and films by adjusting the geometrical parameters of the SiNP system. In addition, we propose a bidirectional deep neural network that can accurately extract the complex relationship between the geometric parameters and color properties. The neural network model achieves nearly perfect accuracy on the predicted colors and achieves high accuracies of 96.56% and 99.34% on the design of geometric parameters of monodisperse and polydisperse SiNPs, respectively, embedded in PMMA. Our work evaluated and designed colored SiNP systems, which will provide opportunities to explore the related applications of SiNP-based materials.

## Figures and Tables

**Table 1:** Inverse design comparison of five randomly selected targets in a polydisperse (*v*_eff_ = 0.01) SiNP system (medium is PMMA).

	Target Geometry Parameters	Target Color		Target *L, a, b*	Design Geometry Parameters	Design *L*′, *a*′, *b*′	Design Color		Δ*E*
Case 1	*r*_eff_ = 50 nm *h* = 5 mm *f*_v_ = 3.0 × 10^−5^	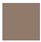	R:144 G:122 B:106	*L* = 52.90 *a* = 5.91 *b* = 12.10	*r*_eff_ = 52.57 nm *h* = 5.74 mm *f*_v_ = 2.58 × 10^−5^	*L*′ = 52.76 *a*′ = 6.79 *b*′ = 10.15	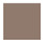	R:144 G:121 B:109	2.138
Case 2	*r*_eff_ = 52 nm *h* = 1 mm *f*_v_ = 1.5 × 10^−5^	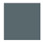	R:89 G:104 B:109	*L* = 42.80 *a* = −4.42 *b* = −4.94	*r*_eff_ = 50.64 nm *h* = 4.05 mm *f*_v_ = 5.2 × 10^−6^	*L*′ = 42.81 *a*′ = −4.41 *b*′ = −4.49	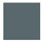	R:89 G:104 B:108	0.443
Case 3	*r*_eff_ = 52 nm *h* = 9.5 mm *f*_v_ = 1.0 × 10^−5^	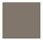	R:128 G:118 B:108	*L* = 50.40 *a* = 2.04 *b* = 6.87	*r*_eff_ = 51.09 nm *h* = 5.74 mm *f*_v_ = 1.47 × 10^−5^	*L*′ = 50.80 *a*′ = 1.23 *b*′ = 7.93	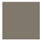	R:129 G:120 B:108	1.391
Case 4	*r*_eff_ = 80 nm *h* = 2 mm *f*_v_ = 8.5 × 10^−5^	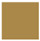	R:169 G:136 B:73	*L* = 58.60 *a* = 4.42 *b* = 38.10	*r*_eff_ = 80.00 nm *h* = 5.88 mm *f*_v_ = 8.1 × 10^−5^	*L*′ = 58.61 *a*′ = 4.43 *b*′ = 38.22	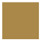	R:169 G:136 B:73	0.1184
Case 5	*r*_eff_ = 100 nm *h* = 2 mm *f*_v_ = 6.0 × 10^−5^	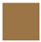	R:156 G:117 B:70	*L* = 52.20 *a* = 9.61 *b* = 31.90	*r*_eff_ = 99.09 nm *h* = 3.34 mm *f*_v_ = 3.6 × 10^−5^	*L*′ = 52.21 *a*′ = 9.61 *b*′ = 31.94	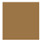	R:156 G:117 B:70	0.046

## Data Availability

The data presented in this study are available upon request from the corresponding author.

## Supplementary Materials

The following supporting information can be downloaded at https://www.mdpi.com/article/10.3390/nano12152715/s1. Figure S1: Variation of chromaticity coordinates with silicon nanoparticle sizes and distributions (host medium: water). Figure S2: Variation of chromaticity coordinates with different host medium (water, PMMA, and PDMS). Figure S3: (a) The simulated reflectance spectra of monodisperse SiNPs embedded in water, PDMS, and PMMA with *h* = 2 mm, *f*_v_ = 1.0 × 10^−5^, and different radii. (b,c) The comparison of *n*_m_ and *κ*_m_ values for the three media. Figure S4: The loss function of the training and validation sets in the forward neural network (a) and inverse neural network (b), respectively. Figure S5: Results of the test samples in the forward network. (a,b) The predicted and simulated chromaticity coordinate values for monodisperse and polydisperse (*v*_eff_ = 0.01) SiNPs embedded in PMMA, respectively. Section S1: Detailed training process of neural network and numerical simulation process. References [39,40,41] are cited in the Supplementary Materials.
