# Supplementary material for: Evaluation and Design of Colored Silicon Nanoparticle Systems Using a Bidirectional Deep Neural Network
Source: Nanomaterials (Basel). 2022 Aug 7;12(15):2715. doi: 10.3390/nano12152715 (PMC9370442; doi:10.3390/nano12152715)
Supplement: Supplementary file 1 [file nanomaterials-12-02715-s001.zip › nanomaterials-1836820-supplementary.pdf]

Supplementary Materials

Evaluation and design of colored silicon nanoparticle systems using a bidirectional deep neural network

Yan Zhou [1,2], Lechuan Hu [1,2], Chengchao Wang [1,2,*] and Lanxin Ma [1,2,*]

[1] School of Energy and Power Engineering, Shandong University, Jinan 250061, China
[2] Optics & Thermal Radiation Research Center, Institute of Frontier and Interdisciplinary Science, Shandong University, Qingdao 266237, China
* Correspondence: sduwcc18@sdu.edu.cn (C.W.); malanxin@sdu.edu.cn (L.M.)

Abstract: Silicon nanoparticles (SiNPs) with lowest-order Mie resonance produce non-iridescent and non-fading vivid structural colors in the visible range. However, the strong wavelength dependence of the radiation pattern and dielectric function makes it very difficult to design nanoparticle systems with the desired colors. Most existing studies focus on monodisperse nanoparticle systems, which are unsuitable for practical applications. This study combined the Lorentz–Mie theory, Monte Carlo, and deep neural networks to evaluate and design colored SiNP systems. The effects of the host medium and particle size distribution on the optical and color properties of the SiNP systems were investigated. A bidirectional deep neural network achieved accurate prediction and inverse design of structural colors. The results demonstrated that the particle size distribution flattened the Mie resonance peak and influenced the reflectance and brightness of the SiNP system. The SiNPs generated vivid colors in all three of the host media. Meanwhile, our proposed neural network model achieved a near-perfect prediction of colors with high accuracy of the designed geometric parameters. This work accurately and efficiently evaluates and designs the optical and color properties of SiNP systems, thus accelerating the design process and contributing to the practical production design of color inks, decoration, and printing.

Keywords: Silicon nanoparticles; Structural color; Lorentz–Mie theory; Deep neural networks; Monte Carlo simulations

	v_{eff} = 0		v_{eff} = 0.01		v_{eff} = 0.05	
r_{eff} = 50 nm		x= 0.264 y= 0.259		x= 0.265 y= 0.287		x= 0.288 y= 0.340
r_{eff} = 60 nm		x= 0.261 y= 0.330		x= 0.287 y= 0.361		x= 0.324 y= 0.377
r_{eff} = 70 nm		x= 0.345 y= 0.470		x= 0.356 y= 0.436		x= 0.359 y= 0.399
r_{eff} = 80 nm		x= 0.425 y= 0.443		x= 0.411 y= 0.433		x= 0.383 y= 0.410
r_{eff} = 90 nm		x= 0.442 y= 0.391		x= 0.428 y= 0.404		x= 0.396 y= 0.401
r_{eff} = 100 nm		x= 0.418 y= 0.371		x= 0.416 y= 0.382		x= 0.397 y= 0.391
r_{eff} = 110 nm		x= 0.383 y= 0.374		x= 0.397 y= 0.367		x= 0.394 y= 0.380
r_{eff} = 120 nm		x= 0.381 y= 0.352		x= 0.382 y= 0.353		x= 0.385 y= 0.371

Figure S1. Variation of chromaticity coordinates with silicon nanoparticle sizes and distributions (host medium: water)

	Water		PMMA		PDMS	
r_{eff} = 50 nm		x= 0.264 y= 0.259		x= 0.287 y= 0.299		x= 0.264 y= 0.260
r_{eff} = 60 nm		x= 0.261 y= 0.330		x= 0.291 y= 0.350		x= 0.263 y= 0.334
r_{eff} = 70 nm		x= 0.345 y= 0.470		x= 0.351 y= 0.438		x= 0.344 y= 0.452
r_{eff} = 80 nm		x= 0.425 y= 0.443		x= 0.399 y= 0.410		x= 0.410 y= 0.424
r_{eff} = 90 nm		x= 0.442 y= 0.391		x= 0.402 y= 0.384		x= 0.415 y= 0.386
r_{eff} = 100 nm		x= 0.418 y= 0.371		x= 0.386 y= 0.386		x= 0.397 y= 0.378
r_{eff} = 110 nm		x= 0.383 y= 0.374		x= 0.380 y= 0.376		x= 0.377 y= 0.375
r_{eff} = 120 nm		x= 0.381 y= 0.352		x= 0.380 y= 0.349		x= 0.376 y= 0.352

Figure S2. Variation of chromaticity coordinates with different host medium (water, PMMA, PDMS)

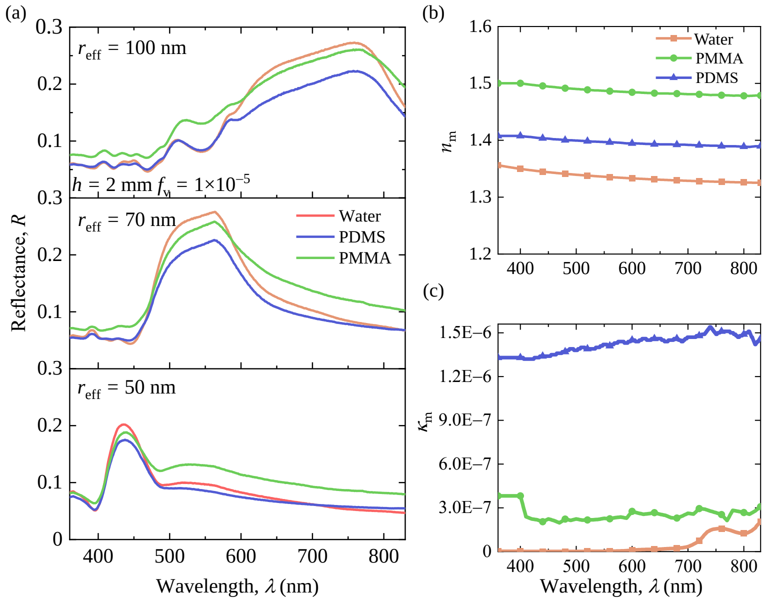

Figure S3. (**a**) The simulated reflectance spectra of monodisperse silicon nanoparticles embedded in water, PDMS, and PMMA with h = 2 mm, f_v = 1.0×10$^{-5,}$ and different radii. (**b**-**c**) The comparison of n_m and κ_m values for the three media [1-3].

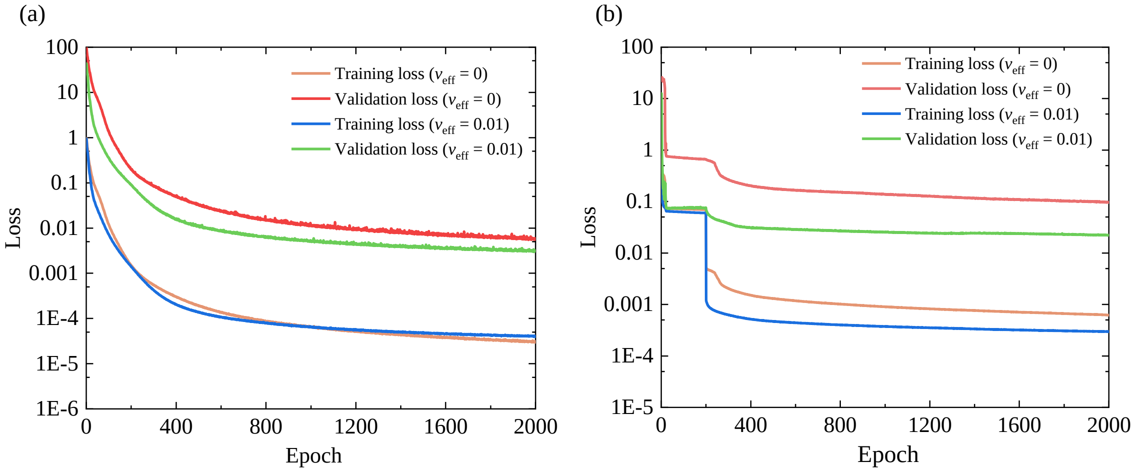

Figure S4. The loss function of the training and validation sets in the forward neural network (**a**) and inverse neural network (**b**), respectively.

(a)

Simulation		Prediction	
	x= 0.287 y= 0.299		x= 0.29 y= 0.30
	x= 0.345 y= 0.348		x= 0.34 y= 0.35
	x= 0.351 y= 0.438		x= 0.35 y= 0.44
	x= 0.409 y= 0.419		x= 0.41 y= 0.42
	x= 0.435 y= 0.394		x= 0.44 y= 0.39
	x= 0.398 y= 0.396		x= 0.40 y= 0.40
	x= 0.392 y= 0.387		x= 0.39 y= 0.39
	x= 0.407 y= 0.360		x= 0.41 y= 0.36

(b)

Simulation		Prediction	
	x= 0.289 y= 0.318		x= 0.29 y= 0.32
	x= 0.346 y= 0.375		x= 0.35 y= 0.38
	x= 0.332 y= 0.378		x= 0.33 y= 0.38
	x= 0.400 y= 0.415		x= 0.40 y= 0.42
	x= 0.427 y= 0.404		x= 0.43 y= 0.40
	x= 0.397 y= 0.387		x= 0.40 y= 0.39
	x= 0.395 y= 0.375		x= 0.40 y= 0.37
	x= 0.393 y= 0.364		x= 0.39 y= 0.36

Figure S5. Results of the test samples in the forward network. (a) and (b) are the predicted and simulated chromaticity coordinate values for monodisperse and polydisperse (v_{eff} = 0.01) SiNPs embedded in PMMA, respectively.

Section S1. Detailed training process of neural network and numerical simulation process

In this work, the process of colors design includes (1) one–time investment of comprehensive simulation data, (2) neural network training and (3) the application of well–trained bidirectional neural network model. In general, the CPU calculation time mainly takes the first numerical simulation process and depends on computing resources. In this work, the calculation time of 13680 sets of parameters spend about 3weeks by using our computer workstation (CPU: Intel Xeon 8173, 56 cores, 112 threads, 2.0GHz basic frequency). For the structure optimization of bidirectional neural network, our PC computer (CPU: Intel Core i5–11400) can complete the training in about half an hour. After finishing the model training, the prediction and inverse design of colors usually finish in several seconds.

The training hyperparameters are listed as follows. Epochs: 2000; batch size: 64; activation function: ReLU; loss function: mean squared error (MSE); optimizer: Adam; learning rate: 0.001; learning rate scheduler: MultiStepLR; milestones = [500, 1000, 1500, 1800] (forward training) and [1800, 1900] (inverse training); gamma = 0.1.

References

1. Segelstein, D.J. The complex refractive index of water. University of Missouri-Kansas City: Kansas City, MO, USA, 1981.
2. Zhang, X.; Qiu, J.; Li, X.; Zhao, J.; Liu, L. Complex refractive indices measurements of polymers in visible and near-infrared bands. *Appl. Opt.* **2020**, *59*, 2337-2344.
3. Zhang, X.; Qiu, J.; Zhao, J.; Li, X.; Liu, L. Complex refractive indices measurements of polymers in infrared bands. *J. Quant. Spectrosc. Radiat. Transf.* **2020**, *252*, 107063.
